# Ploidy‐ and Purity‐Adjusted Allele‐Specific DNA Analysis Using CLONETv2

**DOI:** 10.1002/cpbi.81

**Published:** 2019-06-21

**Authors:** Davide Prandi, Francesca Demichelis

**Affiliations:** ^1^ Department of Cellular, Computational and Integrative Biology (CIBIO) University of Trento Trento Italy; ^2^ Englander Institute for Precision Medicine New York Presbyterian Hospital–Weill Cornell Medicine New York New York; ^3^ Department of BioMedical Research University of Bern Bern Switzerland

**Keywords:** allele‐specific analysis, cancer genomics, clonality, ploidy, purity

## Abstract

High‐throughput DNA sequencing technology provides base‐level and statistically rich information about the genomic content of a sample. In the contexts of cancer research and precision oncology, thousands of genomes from paired tumor and matched normal samples are profiled and processed to determine somatic copy‐number changes and single‐nucleotide variations. Higher‐order informative analyses, in the form of allele‐specific copy‐number assessments or subclonality quantification, require reliable estimates of tumor DNA ploidy and tumor cellularity. CLONETv2 provides a complete set of functions to process matched normal and tumor pairs using patient‐specific genotype data, is independent of low‐level tools (e.g., aligner, segmentation algorithm, mutation caller) and offers high‐level functions to compute allele‐specific copy number from segmented data and to identify subclonal population in the input sample. CLONETv2 is applicable to whole‐genome, whole‐exome and targeted sequencing data generated either from tissue or from liquid biopsy samples. © 2019 The Authors.

## INTRODUCTION

Massive sequencing efforts, such as those of The Cancer Genome Atlas (TCGA) and the International Cancer Genome Consortium (ICGC), have generated a comprehensive collection of sequenced genomes of cancer patients, opening a new era for genomics. Advanced analyses of genomic sequencing data require accurate estimation of DNA cellularity (purity, 1 – DNA admixture) and tumor ploidy to allow appropriate comparative computation. DNA admixture refers to the amount of non‐cancer cells in a tumor sample, whereas ploidy represents the average number of chromosome set in a cell. Human healthy cells are diploid, whereas tumor cells often demonstrate a dramatically variable ploidy number, depending on the tumor type (Chunduri & Storchova, 2019; Danielsen, Pradhan, & Novelli, 2016). The impact of ploidy changes on tumor evolution and prognosis is as yet unclear, but recent pan‐cancer studies have shed some light on this issue. In a primary tumor pan‐cancer cohort from the TCGA project, cell proliferation and immune evasion, two hallmarks of cancer, were deregulated in high‐aneuploidy samples (Davoli, Uno, Wooten, & Elledge, 2017; Taylor et al., 2018). In a pan‐cancer cohort of 9,692 patients with advanced disease, aneuploidy was associated with poor survival (Bielski et al., 2018).

A recent review (Aran, Sirota, & Butte, 2015) highlighted the importance of purity estimation in analyzing sequencing data. For instance, phylogenetic reconstruction of tumor evolution from multisample DNA sequencing data from a single patient stringently relies on the quantification of the variant allelic fraction (VAF) of single‐nucleotide variants (SNV) (Gundem et al., 2015), which is affected by both the DNA admixture (normal cells dilute SNV VAFs) and the ploidy (polyploidy increases the total number of alleles) of each tumor sample. The same issues also affect the determination of the absolute number of copies of a genomic segment in a tumor sample (Carter et al., 2012). Many computational methods identify somatic copy‐number aberrations from the relative amounts of DNA in a tumor and its matched normal sample, but accurate estimation of the integer number of copies of each allele requires purity and ploidy adjustments (Bao, Pu, & Messer, 2014).

These considerations call for the development of computational tools to quantify tumor purity and ploidy. In the pre‐sequencing era, several tools were developed for high‐density single‐nucleotide polymorphism (SNP) array data (e.g., Carter et al., 2012; Van Loo et al., 2010); with these, typically the tumor‐to‐control‐signal log ratio (hereafter logR) and the abundance of allele‐specific signal (B allele frequency, BAF) distributions are jointly analyzed to infer DNA admixture and ploidy. However, array‐based tools are limited by the number of the genomic bases assayed (mainly in the range of 0.5 million to 2 million sites) and by the signal dynamic range. Next‐generation sequencing platforms overcome these limitations while preserving the same data features to exploit (Aran et al., 2015): allelic fraction (AF) of inherited heterozygous SNP loci (hereafter called *informative SNPs*) and sequencing coverage resemble the BAF and logR data of SNP arrays, respectively. The statistically richer data offered by sequencing makes it possible to perform more complex analyses such as allele‐specific copy‐number and clonality estimates.

In general, available methods to estimate ploidy and DNA admixture adopt a global approach, and the distributions of AFs and logR values are conjointly used to infer DNA admixture and ploidy. Intuitively, it is evident that the AF of informative SNPs is distributed around 0.5 in a 100% admixed tumor sample (up to the reference mapping bias; Degner et al., 2009), and lower AFs imply lower DNA admixture. LogR data are used as a covariate, as AF also depends on the number of available alleles. If no tumor cell subpopulations are present (that is, if the copy‐number profile of a tumor sample is homogeneous, i.e., the ratio of subclonal deletions/amplifications is low), global inference approaches capture the DNA admixture content well. However, in the presence of complex genomic events, such as chromothripsis (Stephens et al., 2011) or chromoplexy (Baca et al., 2013), or after multiple treatments that diversify the tumor cell population, global approaches are suboptimal.

CLONET (CLONality Estimate in Tumor; Prandi et al., 2014) is a stand‐alone tool specifically designed with a local approach to clonality estimation to handle heterogeneous tumor samples. Briefly, consider a tumor sample T with a hemizygous deletion HeD and the set of informative SNPs S lying within HeD. The AF value of SNPs in S is the convolution of the AF of the different cell populations composing T. If HeD is subclonal (that is, not all tumor cells harbor this deletion), the tumor sample comprises three main cell populations: (i) non‐tumor cells contributing to DNA admixture, with expected AFs of SNPs in S around 0.5; (ii) tumor cells not harboring HeD, such that the AFs of SNPs in S cannot be distinguished from those of non‐tumor cells; and (iii) tumor cells harboring HeD, in which the AF could either be equal to 1 (if the deleted allele harbors the alternative base) or to 0 (if the deleted allele harbors the reference allele). Based on the observation that apparent DNA admixture is higher in subclonal deletions than in clonal deletions, CLONET estimates DNA admixture at each hemizygous deletion and then identifies the most clonal deletions to finally designate the sample DNA admixture. This results in a more accurate estimation of DNA admixture, which would otherwise be overestimated, in tumors with a significant fraction of subclonal deletions.

Here, we present CLONET version 2 (CLONETv2), an R package (R Core Team, 2017) available at The Comprehensive R Archive Network (https://cran.r‐project.org/) that includes significant improvements over the original CLONET implementation. This is the result of its application to several clinical cohorts, including tissue and plasma samples, and to a variety of sequencing platforms, such as whole‐genome, whole‐exome, and targeted sequencing panels. In Carreira et al. (2014), CLONET was used to estimate DNA admixture from a custom sequencing panel of ∼40 kb designed to analyze circulating tumor DNA of plasma samples from metastatic patients, and the algorithm was modified to improve sensitivity in samples with <10% tumor cells. In Beltran et al. (2016), CLONET was extended to provide allele‐specific copy‐number data from whole‐exome sequencing experiments; for each genomic segment in each study cohort tumor, the study reports the number of copies of each allele using ploidy, DNA admixture, logR, and the AF of informative SNPs. In Faltas et al. (2016), the clonality analysis capability of CLONET was improved to account for complex allele‐specific combinations and SNVs. Since its initial conception and application to whole‐genome sequencing data (Baca et al., 2013; Prandi et al., 2014), CLONET improvements have been used in several studies (including Beltran et al., 2015; Boysen et al., 2015; Cancer Genome Atlas Research Network, 2015; and Mu et al., 2017). Here, we present a documented version of CLONETv2 to uniformly highlight the approach features and propose it as an R package to make the tool available to a broader audience.

## Basic Protocol 1 COMPUTING BETA TABLE

**Figure 1:**
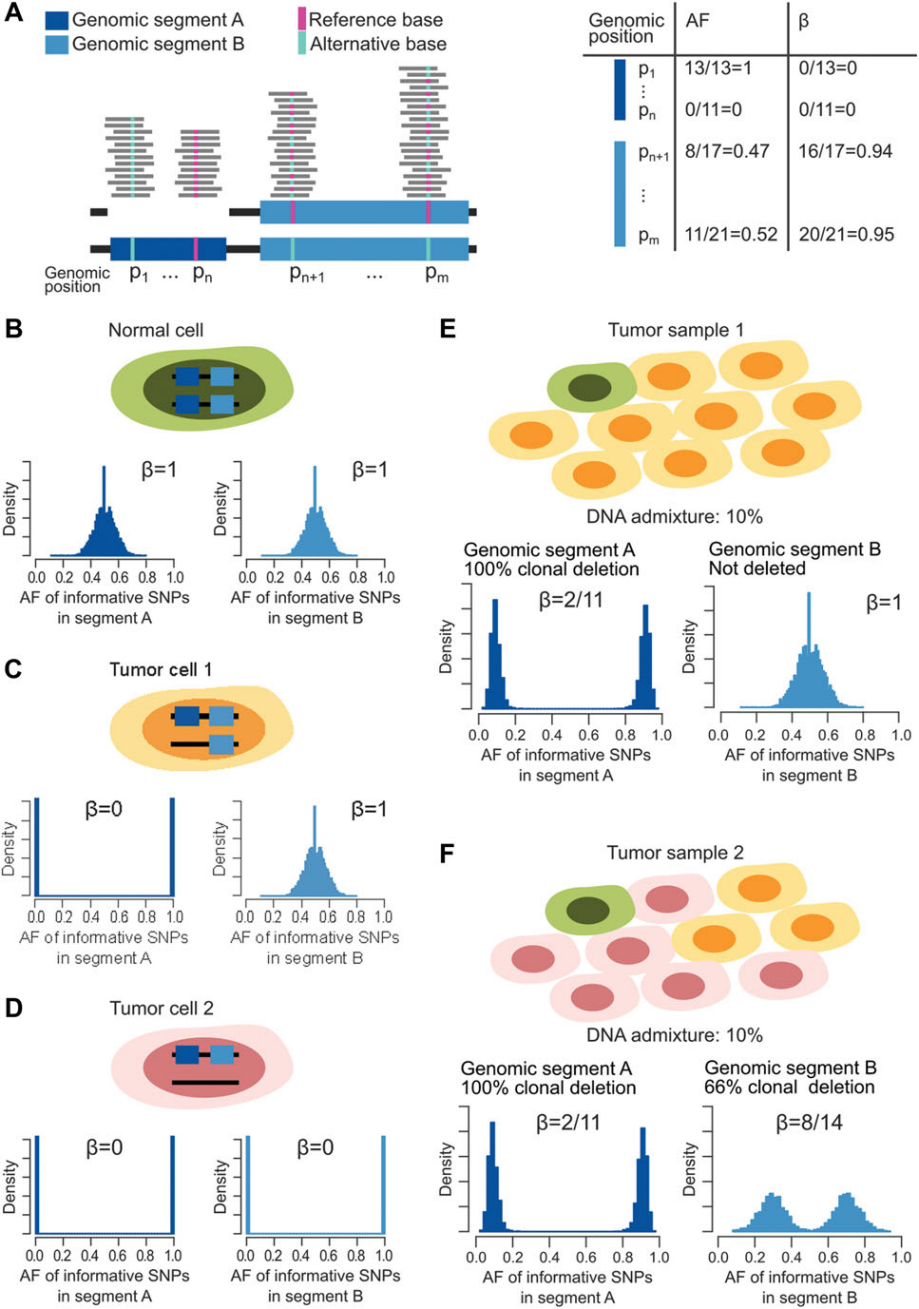
Cartoon of the computation of beta and allelic fraction of informative SNPs. (**A**) Example of the allelic fraction (AF) and beta (β) values computed for five genomic positions (p_1_ to p*_m_*) corresponding to five informative SNPs. Positions p_1_ to p*_n_* are within a hemizygously deleted genomic segment, A, whereas genomic positions p*_n_*_+1_ to p*_m_* lie within a wild‐type genomic segment, B. (**B** to **D**) Examples of a normal cell and two different tumor cells. Tumor cells 1 and 2 differ in the status of genomic segment B. Histograms below the cell cartoons report the expected distribution of the AF of SNPs in genomic segments A and B together with the associated beta values. (**E** and **F**) Examples of two different tumor samples. Tumor sample 1 includes one normal cell and nine tumor cells with deleted genomic segment A and wild‐type genomic segment B. Tumor sample 2 differs from tumor sample 1 in the presence of six tumor cells with a hemizygous deletion of genomic segment B. Expected distribution of the AF of informative SNPs together with estimated beta are depicted below each tumor sample cartoon.

**Figure 2:**
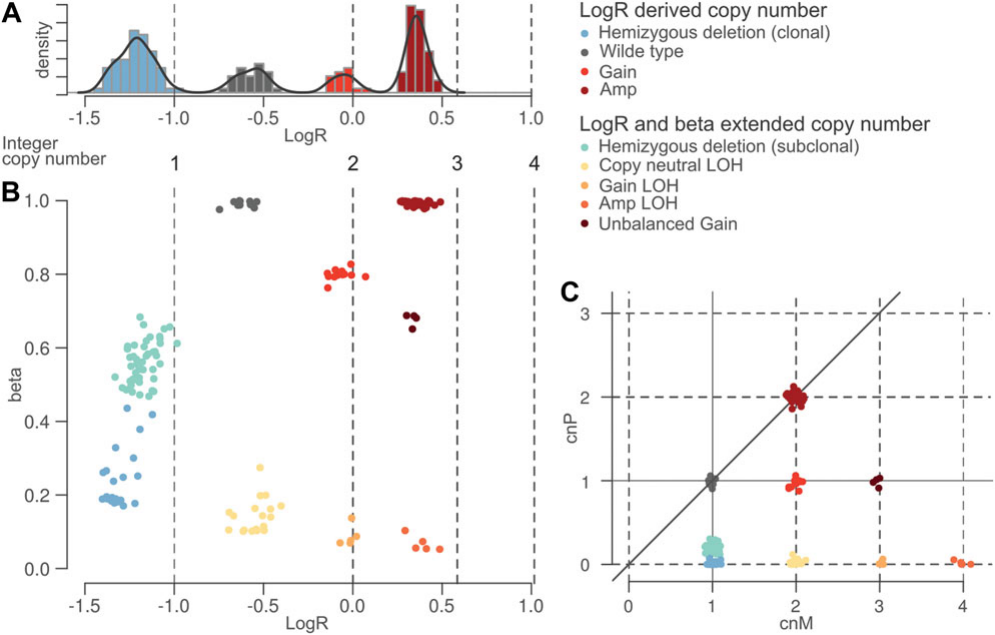
Sketch of CLONETv2 copy‐number‐space transformations. (**A**) Example of histogram and density plots of the distribution of logR signal in a tumor sample. Expected positions of integer copy numbers in a diploid 100% pure tumor sample are listed below. (**B**) Expansion of the monodimensional logR signal of panel A in the two‐dimensional beta‐vs.‐logR space. Each dot represents a genomic segment, and vertical dashed lines correspond to integer copy number as in panel A. Color code clusters genomic segments with homogenous copy number. (**C**) Allele‐specific copy‐number projection of the beta vs. logR data of panel B. Each dot represents a genomic segment with maternal copy‐number allele cnM and paternal copy‐number allele cnP. Maternal and paternal alleles are assigned arbitrarily. The color code is consistent with that in panel B.

All reads of a human DNA next‐generation sequencing experiment that map within a genomic segment derive from either one of the parental chromosomes of origin. Reads can be split into two sets: a *copy‐number‐neutral* set that contains equal numbers of reads from the maternal and paternal chromosomes, and an *active reads* set that includes sequences from only one parent. Generally speaking, given two random reads, it is impossible to determine whether or not they represent the same allele; however, if the two reads span an informative SNP, the allele of origin can be identified. For reads over informative SNPs, the number of reads (local coverage) supporting the reference or the alternative SNP represents the number of copies and the origin of the alleles present in the tumor sample. Each informative SNP can be characterized by its allelic fraction (AF), which depends on the genomic context. For instance, let us consider the two informative SNPs within a monoallelic deletion of the genomic segment denoted A in Figure 1A. At position p_1_, only the alternative allele is present and AF = 1, whereas at position p*_n_*, the alternative allele is deleted and AF = 0. In contrast, in the wild‐type genomic segment B, the AF values of informative SNPs at positions p*_n_*_+1_ and p*_m_* are distributed around 0.5, as both alleles contribute equally to the local coverage. Now, the percentage of neutral reads (known as beta, β) at p_1_ and p*_n_* is equal to 0, regardless of which allele is deleted, whereas at wild‐type genomic positions, p*_n_*_+1_ and p*_m_* each approximate 1, as no active reads are present. Overall, SNPs within somatically aberrant segments are easier to characterize using the beta values as compared to the AFs, as the former is independent from the deleted allele. In a heterogeneous tumor sample, the distributions of AFs and betas result from the convolution of the distribution observed in basic wild‐type and monoallelic deleted segments. As an example, Figure 1B depicts the distribution of the AF and the associated beta of the informative SNPs in genomic segments A and B in the case of a normal cell, whereas Figures 1C and 1D show how the distributions change in tumor cells with monoallelic deletion of only genomic segment A, or of both A and B, respectively. Figure 1E represents the case of a tumor sample with one normal cell (Fig. 1B) and nine tumor cells 1 (Fig. 1C). The DNA admixture is 1/10, and the AF could assume values around 1/11 or 10/11, whereas beta is 2/11. Genomic segment B is not deleted, and therefore the AF and the beta are as in the normal cell. Figure 1F represents a more complex situation involving one normal cell (Fig. 1B), three “tumor cells 1” (Fig. 1C), and six “tumor cells 2” (Fig. 1D). The AF and beta of informative SNPs in genomic segment A are as in Figure 1E, but only the six tumor cells 2 carry the monoallelic deletion of genomic segment B. In this case, the AF distribution modes are centered on 4/14 and 10/14, depending on the depleted base, whereas beta is 8/14. The full characterization of beta is described by Prandi et al. (2014), and in Beltran et al. (2016) we defined CLONET master equations that describe allele‐specific copy number of maternal and paternal alleles, cnM and cnP, as a function of the percentage of neutral reads beta, the log_2_ ratio values adjusted by ploidy logRp, and the DNA admixture *G*, as:

(1) cnM =(2− beta )( beta 2 logRp −G)+2G(1− beta )1−G beta  cnP = beta 2 logRp −G1−G

where maternal and paternal allele are arbitrarily assigned. Figure 2 sketches the transformation of the log_2_ ratio space implied by Equation 1. Figure 2A reports the histogram of the log_2_ ratio signal in a tumor sample: peaks in the distribution correspond to different copy‐number states, whereas deviations from the position of the expected peaks (below) depend on ploidy and DNA admixture values. It is difficult to identify the peak that corresponds to wild‐type segments using only log_2_ ratio signal. When we expand the monodimensional logR space with beta (Fig. 2B), segments that contribute to the same peak along the logR dimension form different clusters in the beta‐vs.‐logR space. Of note, the beta‐vs.‐logR plot still reflects ploidy and DNA admixture, whereas the cnM and cnP space (see Equation 1) allows straightforward interpretation of the copy number and clonality status of each genomic segment.

The function compute_beta_table estimates the beta of a genomic segment as described in Carreira et al. (Carreira et al., 2014). The function compute_beta_table includes the following input:

- seg_tb: a table resulting from DNA segmentation; for each genomic segment, the table reports chromosome, start/end position and the log_2_ ratio of the tumor over the normal coverage, as defined in the Circular Binary Segmentation algorithm (Olshen, Venkatraman, Lucito, & Wigler, 2004);
- pileup_normal, pileup_tumor: two tables reporting allelic fraction and coverage of SNPs in normal and matched tumor samples, respectively; for each SNP, each table reports genomic coordinates (chromosome and position), allelic fraction, and coverage;
- min_af_het_snps, max_af_het_snps: for each SNP in the pileup_normal table, set minimum and maximum allelic fraction to consider the SNP as informative;
- min_required_snps: the minimum number of informative SNPs in a genomic segment from seg_tb to retain the segment;
- min_coverage: the minimum mean coverage of informative SNPs to retain a segment.

As output, the function compute_beta_table extends the input table seg_tb. For each segment in seg_tb, the function compute_beta_table returns the following values:

- beta: estimated value for the input segment;
- nsnps: number of informative SNPs in the input segment;
- cov: mean coverage of informative SNPs in the input segment;
- n_beta: estimated value for the input segment considering the matched normal sample. This value is expected to be 1, except in the case of germline copy‐number variation or sequencing‐related errors.

The interpretation of the function compute_beta_table output is not an easy task due to the identifiability problem — i.e., the fact that more than one combination of ploidy and DNA admixture fit the observed data (Li & Xie, 2014). However, upon definition of ploidy and DNA admixture, Equation 1 completely defines the absolute copy numbers of both alleles. We will exploit this capability in Support Protocol 2, where Equation 1 is used to plot the expected beta and logR ratio against estimated values. The optional parameter plot_stats of the compute_beta_table function plots useful summary statistics for a "sanity check" of the output. In particular, when plot_stats is TRUE, the function returns:

- number of processed segments: the number of segments in the input seg_tb table;
- number of segments with a valid beta estimate: the number input segments for which beta value is computed; this value is affected by the number of informative SNPs and their mean coverage;
- quantiles of input segment lengths: the quantiles of the distribution of the length of the input segments; the expected distribution depends on the segmentation algorithm used to produce the seg_tb table, but in general small values result in a low number of informative SNPs, whereas large segments may indicate undersegmentation that in turn affects beta estimates;
- quantiles of informative SNPs input segment coverage: the quantiles of the distribution of the mean coverage of the input segments; expected coverage depends on the sequencing experiment, but a low value may indicate problems with the input sample;
- quantiles of number of informative SNPs per input segment: the quantiles of the distribution of the number of informative SNPs in the input segments; expected number of informative SNPs per kb is ∼0.33 (based on common SNPs), and therefore, this value combined with input segment lengths gives information about the quality of the pileup data.

### Necessary Resources

#### Hardware

64‐bit computer running Linux with ≥8 GB RAM

#### Software

The library has been tested with R version 3.5.2 and the R libraries parallel 3.5.2, ggplot2 3.1.0, sets 1.0‐18, arules 1.6‐3, and ggrepel 0.8.0

1 Prepare tumor and normal pileups as described in Support Protocol 1 or with other computational tools. The output of this step comprises two files, tumor.pileup and normal.pileup.
2 Prepare tumor segmented data in the file tumor_segments.txt with columns compatible with the parameter seg_tb described above.
3 Run R from the command line:
$ R
4 Install CLONETv2 for the first time:
> install.packages(“CLONETv2”)
5 Load the library:
> library(CLONETv2)
6 Load input files:
> seg_tb <‐ read.table(system.file(“sample.seg”, package = “CLONETv2”),header = T, as.is=T)
> pileup_tumor <‐ read.table(system.file(“sample_tumor_pileup.tsv”, package = “CLONETv2”),header = T, as.is=T)
> pileup_normal <‐ read.table(system.file(“sample_normal_pileup.tsv”, package = “CLONETv2”),header = T, as.is=T)
7 Compute beta for each input segment with default parameters:
> bt <‐ compute_beta_table(seg_tb, pileup_tumor, pileup_normal)
8 Compute beta activating the plot_stats parameter:
> bt <‐ compute_beta_table(seg_tb, pileup_tumor, pileup_normal, plot_stats=T)

This results in the following output:

Computed beta table of sample “sample1”
Number of processed segments: 65
Number of segments with valid beta: 49 (75%)
Quantiles of input segment lengths:
0%: 2860
25%: 17504185
50%: 38004799
75%: 59311449
100%: 147311449
Quantiles of input segment coverage:
0%: 47.0000
25%: 137.7893
50%: 168.3820
75%: 186.6769
100%: 695.6145
Quantiles of number of informative SNPs per input segment:
0%: 0
25%: 12
50%: 99
75%: 213
100%: 404

## Support Protocol 1 PREPARING PILEUP DATA

This protocol describes the steps used to prepare pileup data from a set of SNPs and matched tumor and normal .bam (BAM) files (Li et al., 2009). The tables pileup_normal and pileup_tumor report allelic fraction and coverage for a set of SNP positions. Candidate SNP positions can be downloaded directly from the dbSNP FTP server (ftp://ftp.ncbi.nlm.nih.gov/snp/). We suggest starting from the largest possible set of SNPs, as the larger the number of informative SNPs, the more reliable the CLONETv2 estimates. Pileups from BAM files can be obtained using any of several tools. Here we describe how to prepare pileups using ASEQ (Romanel, Lago, Prandi, Sboner, & Demichelis, 2015), a tool freely available at http://demichelislab.eu/tools/ASEQ.

### Necessary Resources

#### Hardware

64‐bit computer running Linux with ≥8 GB RAM

#### Software

ASEQ, curl

#### Input files

BAM files tumor.bam and normal.bam containing aligned reads from genomic sequencing experiments of matched tumor and normal DNA samples, respectively
VCF (Degner et al., 2009) file known_snp_positions.vcf reporting known SNP positions; ASEQ requires that the input VCF only lists SNPs, i.e., columns ALT and REF must contain one of the values A, C, G, or T. ASEQ parameters include:

- mrq: minimum read quality (ASEQ does not consider as part of the pileup reads with read quality < mrq);
- mbq: minimum base quality (ASEQ does not consider as part of the pileup bases with quality < mbq);
- mdc: minimum depth of coverage (ASEQ output only reports positions with coverage ≥ mdc);
- threads: number of threads available for ASEQ computation.

1 Download and uncompress the last version of ASEQ:
$ curl http://demichelislab.unitn.it/lib/exe/fetch.php?media=aseq‐v1.1.11‐linux64.tar.gz > aseq‐v1.1.11‐linux64.tar.gz
$ tar xvf aseq‐v1.1.11‐linux64.tar.gz

ASEQ code will be available in the subfolder binaries/linux64/.

2 Download and uncompress ASEQ examples:
$ curl http://demichelislab.unitn.it/lib/exe/fetch.php?media=aseq‐examples.tar.gz > aseq‐examples.tar.gz
$ tar xvf aseq‐examples.tar.gz

ASEQ examples will available in the subfolder examples/VCF_samples/.

3 Run ASEQ on example data 1:
$./binaries/linux64/ASEQ mode=PILEUP vcf=examples/VCF_samples/sample1.vcf bam=examples/BAM_samples/sample1.bam mbq=20 mrq=20 mdc=1 threads=1 out=.

ASEQ produces the file sample1.PILEUP.ASEQ, reporting allelic fraction and read coverage from the BAM file sample1.bam, for each position in the VCF file sample1.vcf. The parameters mbq = 20 and mrq = 20 tell ASEQ to ignore, respectively, bases and reads with quality <20. The parameter mdc = 1 instructs ASEQ to ignore positions in the BAM file with no reads. The parameters and the format of the output file .PILEUP.ASEQ are compatible with pileup data required in Basic Protocol 1.

## Basic Protocol 2 COMPUTING PLOIDY

**Figure 3:**
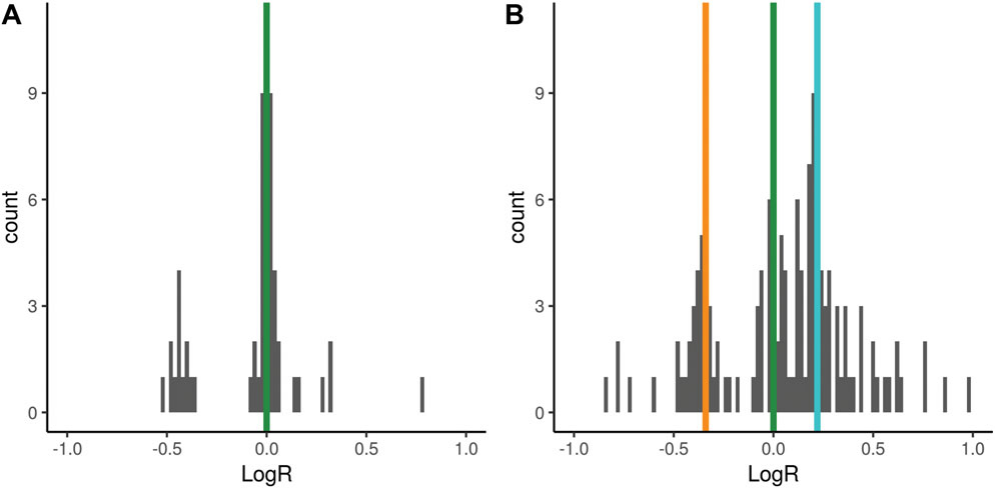
Examples of diploid and aneuploid sample. Histograms of the logR of a diploid tumor sample (**A**) and an aneuploid tumor sample (**B**) are shown. Green line, expected value; orange line, value corresponding to wild‐type segments; turquoise line, value corresponding to segments with copy number 4.

Segmentation algorithms partition input genomic space into segments with homogenous coverage. Given a pair of matched tumor and normal samples, the logR value of a genomic segment is the log_2_ of the ratio between the tumor coverage and the normal sample coverage within the segment. To account for different mean coverage in different sequencing experiments, logR is normalized over the ratio between the mean tumor and the mean normal coverage; this applies both to whole‐genome and whole‐exome data. In the case of higher coverage in the tumor sample, if without normalization the ratio between the mean tumor and the mean normal coverage is *X*, a wild‐type segment would have logR = log_2_(*X*), whereas the expected value is 0 (i.e., same number of alleles between tumor and normal samples). The normalization would, however, introduce a bias whenever the difference in mean coverage between the tumor and the normal sample was due to the presence of an abnormal number of alleles in the tumor (aneuploid) genome. In this case, the normalization leads to a shift in the logR signal. Figure 3A shows an example of a diploid genome sample with 127× and 69× mean tumor and mean normal coverage, respectively. The logR signal is centered on 0, as expected (green line). Figure 3B highlights a more complex case: tumor and normal mean coverage are comparable (125× and 117×, respectively), but the position of the wild‐type segments (orange line) is shifted with respect to the expected value (green line). The shift is representative of the total number of alleles in the genome, and ploidy can be estimated as:

(2) ploidy =2×2−log2 logR  shift

The proof (Equation 2) is reported in the paper originally describing CLONET (Prandi et al., 2014). The example in Figure 3A has a logR shift of 0 and ploidy of 2, whereas the example in Figure 3B has a logR shift of –0.34 and a ploidy of 2.53.

The function compute_ploidy builds on this definition and is implemented to identify wild‐type genomic segments and to estimate how far the logR values deviate from 0. The key step in the search is to restrict the genomic segments space to those with beta = 1, i.e., those with an equal number of maternal and paternal copies. In Figure 3B, this step excludes segments with logR around 0, as their beta is significantly lower than 1 and represent segments with copy number 3 (see Basic Protocol 4). In this context, the green line in Figure 3B is centered on wild‐type segments, and the turquoise vertical line identifies segments of copy number 4. The function compute_ploidy includes the following input parameters:

- beta_table: a table created using the function described in Basic Protocol 1;
- max_homo_dels_fraction (default 0.05): homozygous deletions can provide a confounding factor in the determination of sample ploidy; the parameter sets a percentage of genomic segments that will not be used for ploidy computation as putative homozygous deletion, and overestimating this value does not affect ploidy computation;
- beta_limit_for_neutral_reads (default 0.90): in theory, neutral reads correspond to beta = 1, but experimental noise lowers this value; therefore only segments with beta above the limit are used to compute ploidy;
- min_coverage (default 20): only genomic segments with average coverage at least min_coverage are used to compute DNA admixture;
- min_required_snps (default 10): only genomic segments covering at least min_required_snps informative SNPs are considered for DNA admixture computation.

The function returns the ploidy for the input sample.

### Necessary Resources

#### Hardware

64‐bit computer running Linux with ≥4 GB RAM

#### Software

The library has been tested with R version 3.5.2 and R libraries parallel 3.5.2, ggplot2 3.1.0, sets 1.0‐18, arules 1.6‐3, ggrepel 0.8.0.

1 Run R from the command line:
$ R
2 Compute beta table as described in Basic Protocol 1.
3 Compute ploidy from beta table bt:
> pl <‐ compute_ploidy(bt)

## Basic Protocol 3 COMPUTING DNA ADMIXTURE

DNA admixture is defined as the percentage of non‐tumor cells in a tumor sample. DNA admixture is an important confounding factor in genomic analysis, as it dilutes somatic aberration signal across all genomic and molecular alterations. Relevant to genomic analyses, it dilutes somatic copy‐number aberration (SCNA) and SNV signal. In a 100% pure tumor sample, the expected coverage across a monoallelic (i.e., hemizygous) deletion should be about half of coverage of wild‐type segments, and therefore the logR should be equal to –1 (i.e., log_2_(½)). However, if the purity is 50%, then only half of the total number of cells harbor the hemizygous deletion, and the expected logR is log_2_(¾), or around –0.415. Similarly, the value of beta of a genomic segment varies depending on the level of DNA admixture. In Basic Protocol 1, we saw that the beta of a hemizygous deletion in a 100% pure sample is 0, as no neutral reads are present. However, 50% admixture would increase beta to ⅔, as for each tumor active read there would be two neutral read from the admixed cells. The original CLONET manuscript (Prandi et al., 2014) describes the equations that define the expected logR and beta corresponding to the spectrum of tumor admixture. The function compute_dna_admixture searches the (logR, beta) space defined by the function compute_beta_table (Basic Protocol 1) for a value of DNA admixture that better explains the observed value in the beta_table. The function compute_dna_admixture also requires the ploidy value, as computed by the function compute_ploidy (Basic Protocol 2), to account for the shift in logR values due to possible aneuploidy tumor genomes. The function compute_dna_admixture has the following input parameters:

- beta_table: a table created using the function described in Basic Protocol 1;
- ploidy_table: a table created using the function described in Basic Protocol 2;
- min_coverage (default 20): only genomic segments with average coverage at least min_coverage are used to compute DNA admixture;
- min_required_snps (default 10): only genomic segments covering at least min_required_snps informative SNPs are considered for DNA admixture computation;
- error_tb: the number of informative SNPs and the coverage of the considered segment affect the accuracy of the estimation of beta of a genomic. The table error_tb reports, for each combination of number of informative SNPs and coverage, the expected error around the beta estimate. CLONETv2 embeds a pre‐computed error_tb (details in Prandi et al., 2014) previously tested in several studies (Beltran et al., 2015; Beltran et al., 2016; Faltas et al., 2016). However, specific experimental settings, such as ultra‐deep targeted sequencing or low‐pass whole‐genome sequencing, may require an ad hoc error_tb table.

The function returns the estimated DNA admixture for the input sample as well as minimum and maximum DNA admixture values accounting for errors around beta estimates.

### Necessary Resources

#### Hardware

64‐bit computer running Linux with ≥4 GB RAM

#### Software

The library has been tested with R version 3.5.2 and the R libraries parallel 3.5.2, ggplot2 3.1.0, sets 1.0‐18, arules 1.6‐3, and ggrepel 0.8.0

1 Run R from the command line:
$ R
2 Compute beta table as described in Basic Protocol 1.
3 Compute ploidy table as described in Basic Protocol 2.
4 Given beta table bt and ploidy pl, compute DNA admixture:
> adm <‐ compute_dna_admixture(bt, pl)

## Support Protocol 2 VISUALIZING AND INTERPRETING BETA TABLE, PLOIDY, AND DNA ADMIXTURE

**Figure 4:**
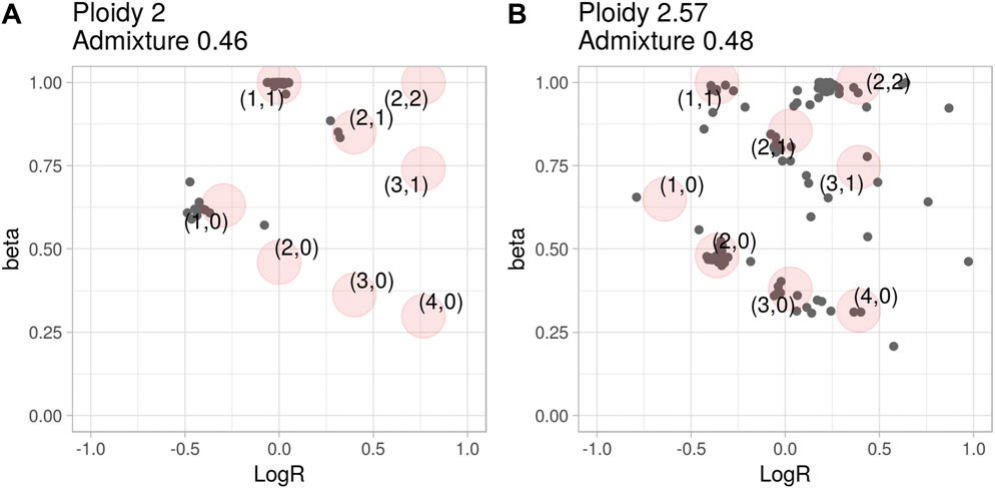
Examples of beta‐vs.‐logR space. Panels **A** and **B** extend the logR histograms of Figure 1A and B, respectively, to the beta‐vs.‐logR space. Each gray dot represents a genomic segment. Large light‐red circles represent expected (beta, logR) values corresponding to the estimated ploidy and DNA admixture (reported above the corresponding plot). A circle corresponding to clonal homozygous deletions, if represented, would be at coordinate (–∞, 1).

Basic Protocol 1 describes how to derive the value of beta for a genomic segment. A tumor sample is then described as a set of (beta, logR) values extending the usual logR space and enabling the computation of ploidy and DNA admixture in Basic Protocols 2 and 3, respectively. To help interpreting the results of Basic Protocols 1 to 3, CLONETv2 provides the function check_ploidy_and_admixture that plots beta‐vs.‐logR space for a given samples. Figure 4A and 4B show the values of beta against the logR of the same samples presented in Figure 3A and B**,** respectively. For each genomic segment, the plot reports the logR as well as the beta computed by function compute_beta_table. To help the user, the function predicts expected (beta, logR) given the input ploidy and DNA admixture level according to the equations described in CLONET paper (Prandi et al., 2014). Predicted values are computed for different combinations of allele‐specific copy numbers (see Basic Protocol 4) and represented as red circles. Comparing the expected (red circles) and the observed (gray dots) values helps the interpretation of the estimates. For instance, segments with logR near 0 in Figure 3B cannot be wild type, as their betas are ∼0.8, a value compatible with the presence of three DNA copies.

### Necessary Resources

#### Hardware

64‐bit computer running Linux with ≥4 GB RAM

#### Software

The library has been tested with R version 3.5.2 and the R libraries parallel 3.5.2, ggplot2 3.1.0, sets 1.0‐18, arules 1.6‐3, and ggrepel 0.8.0

1 Run R from the command line:
$ R
2 Follow Basic Protocols 1, 2, and 3 to compute beta table bt, ploidy table pl, and DNA admixture table adm, respectively.
3 Compute basic beta‐vs.‐logR plot:
> check_plot <‐ check_ploidy_and_admixture(bt,pl,adm)
check_plot is a ggplot object (Wickham, 2009) that can be customized by the user (e.g., for font size, color, line width).
4 Print final plot with the command:
> print(check_plot)

## Basic Protocol 4 COMPUTING ALLELE‐SPECIFIC COPY NUMBER

**Figure 5:**
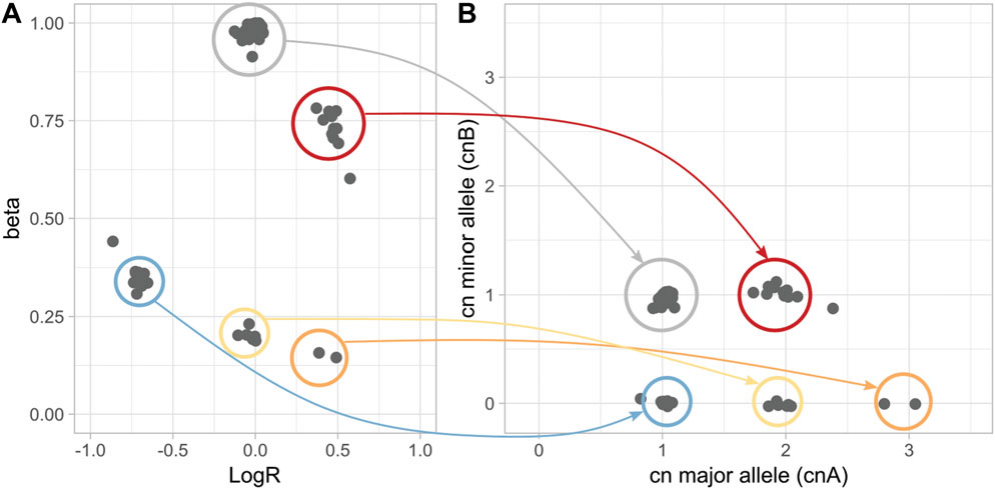
From beta‐vs.‐logR to allele‐specific copy‐number space. (**A**) Beta vs. logR of a tumor sample (as in Fig. 2). (**B**) Allele‐specific plot obtained by transforming the tumor sample data from **A**. Each dot corresponds to a genomic segment for which the copy‐number values of the two alleles are reported (with higher copy‐number values conventionally reported in the *x* axis). Colored arrows and circles show how combinations of beta and logR correspond to different allele‐specific copy‐number values. Color code: gray, wild type; light blue, hemizygous deletion; red, gain; yellow, CN‐LOH (copy‐neutral loss of heterozygosity); orange, gain‐LOH (loss of heterozygosity in which one allele is lost and the logR indicates a gain of DNA).

Figure 3 suggests a relation between the values (beta, logR) for a genomic segment and its allele‐specific copy number. Consider a 100% pure tumor sample and a genomic segment with wild‐type logR, in which the log_2_ ratio is equal to 0; then beta could either be equal to 1 (if one copy each of the maternal and paternal alleles are present) or be equal to 0 (if the two alleles come from the same parent: the copy‐neutral loss of heterozygosity, or CN‐LOH, case). The approach is generalized in Beltran et al. (2016) by defining the exact equations that relates (logR, beta) to allele‐specific copy number, given the ploidy and the DNA admixture. Figure 5A shows an example in which CLONETv2 identifies three classes of loss of heterozygosity: the well‐characterized classes of hemizygous deletion and CN‐LOH, and the less common gain‐LOH, in which one allele is lost but the total copy number (logR value) is consistent with a gain of DNA. Mapping (logR, beta) space to allele‐specific copy‐number space (Fig. 5B) simplifies interpretation the genomic landscape of a sample. Of note, the allele‐specific copy‐number signal in Figure 5B does not contain information about the ploidy and purity of the original sample, making it easy to compare samples with different ploidy and purity values. The example highlights the novelty and power of allele‐specific copy‐number analysis. The function compute_allele_specific_scna_table transforms (logR, beta) pairs into allele‐specific copy‐number pairs (cnA, cnB). The function requires estimates of purity and ploidy and has the following parameters:

- beta_table: a table created using the bt function described in Basic Protocol 1;
- ploidy_table: a table created using the pl function described in Basic Protocol 2;
- admixture_table: a table created using the adm function described in Basic Protocol 3;
- error_tb: the same error_tb used in the function compute_dna_admixture of Basic Protocol 3, step 4;
- allelic_imbalance_th (default 0.5): function compute_allele_specific_scna_table also returns integer values cnA.int and cnB.int for cnA and cnB, respectively. The value cnA.int is the rounded‐off value of cnA if |cnA.int ‐ cnA| < allelic_imbalance_th; otherwise cnA.int is not defined. cnB.int is defined similarly with respect to cnB.

The function compute_allele_specific_scna_table extends input beta_table with columns related to allele‐specific copy‐number:

- log2.corr: logR value adjusted by ploidy and purity: i.e., the logR value the segment would have in a diploid 100% pure tumor sample;
- cnA, cnB: number of copies of major (cnA) and minor (cnB) allele; the values do not contain information about ploidy and purity — indeed, cnA + cnB equals 2 × 2^log2.corr^;
- cnA.int, cnB.int: integer number of copies of major and minor alleles, respectively.

### Necessary Resources

#### Hardware

64‐bit computer running Linux with ≥4 GB RAM

#### Software

The library has been tested with R version 3.5.2 and the R libraries parallel 3.5.2, ggplot2 3.1.0, sets 1.0‐18, arules 1.6‐3, and ggrepel 0.8.0

1 Run R from the command line:
$ R
2 Follow Basic Protocols 1, 2, and 3 to compute beta table bt, ploidy table pl, and DNA admixture table adm, respectively.
3 Given beta table bt, ploidy table pl, and DNA admixture table adm, compute the allele‐specific SCNA table:
> as_tb <‐ compute_allele_specific_scna_table(bt, pl, adm)

## Basic Protocol 5 COMPUTING SOMATIC COPY‐NUMBER CLONALITY

**Figure 6:**
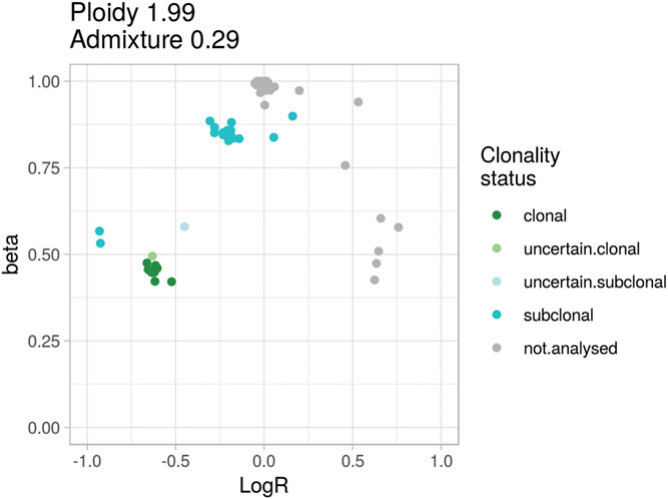
Example of tumor sample with subclonal copy number. Plot of beta vs. logR of a tumor sample with subclonal copy‐number segments. Each dot represents a genomic segment, and the color code indicates clonality status as indicated in the color legend.

A somatic aberration is clonal if all the tumor cells harbor the aberration. Suppose a 100% pure tumor sample with monoallelic deletions of genomic segments D_1_ and D_2_, with 100% and 50% clonality, respectively: i.e., all tumor cells harbor D_1_ deletion, but only 50% harbor D_2_ deletion. The expected logR is then log_2_(½) = –1 for D_1_ and log_2_(¾) (about –0.415) for D_2_. Note that the expected logR for D_2_ is the same that would result given a clonal deletion in a 50% pure sample (see Basic Protocol 3). This is because, in genomic region D_2_, the reads sequenced from cells not harboring the deletion cannot be distinguished from those derived from admixed non‐tumor cells. The same consideration holds for the expected proportion of neutral reads, beta. The CLONET equations (Carreira et al., 2014) build on this intuition and define a map from (logR, beta) pairs to the clonality of somatic copy‐number aberrations. However, fluctuations in the level of coverage that introduce noise in the logR signal, as well as limitations in the sensitivity of the inference of beta due to the number of available informative SNPs, make it difficult to compare the clonality levels of aberrations across different tumor samples. To facilitate such clonality comparisons, the function compute_scna_clonality_table returns a minimum and maximum estimated clonality value and a discretized clonality status. The function considers DNA admixture level, distribution of logR values, and errors around beta estimates and assigns to each genomic segment a minimum and a maximum observed clonality. Lower and upper bound for clonality are used to assign to define the segment clonality status, among *clonal*, *uncertain.clonal*, *uncertain.subclonal*, *subclonal*, and *not.analysed*. Clonal and subclonal statuses correspond to more reliable clonality calls, whereas an uncertain prefix is used when clonality estimate can be affected by the noise of the input data. For instance, Figure 6 reports the example of a tumor sample with two clusters of hemizygous deletions: clonal in (–0.6, 0.45) and subclonal in (–0.25, 0.8). Segments in (–0.9, 0.53) correspond to a region with subclonal homozygous deletion, in which 20% of the tumor cells lack both alleles whereas the other 80% retain one allele. Uncertain clonality status calls refer to segments at (–0.45, 0.58) and at (–0.63, 0.51); compared to clonal segments, the former shows markedly different beta but borderline logR (uncertain.subclonal), and the latter shows only small deviation in beta (uncertain.clonal segment). Not.analysed segments include wild‐type segments and aberrant segments with (logR, beta) values that do not fit CLONETv2 model. The function compute_scna_clonality_table takes a beta table and the associated estimates of purity and ploidy together with the following parameters:

- beta_table: a table created using the function described in Basic Protocol 1;
- ploidy_table: a table created using the function described in Basic Protocol 2;
- admixture_table: a table created using the function described in Basic Protocol 3;
- error_tb: same error_tb used in the function compute_dna_admixture of Basic Protocol 3; error around beta is propagated to clonality estimate and used in its discretization;
- clonality_threshold (default = 0.85): the function compute_scna_clonality_table returns minimum and maximum clonality for input genomic segments; clonality_threshold is used to discretize clonality as described by Prandi et al. (2014);
- beta_threshold (default = 0.9): input beta values below beta_theshold are marked as potentially aberrant and used for clonality estimates.

The function compute_scna_clonality_table extends input beta_table with clonality‐related columns:

- clonality: real value representing the estimated percentage of tumor cells with uniform copy number for a given genomic segment;
- clonality.min, clonality.max: real values representing minimum and maximum estimated clonality given the distribution of beta and logR values;
- clonality.status: discretized clonality.

### Necessary Resources

#### Hardware

64‐bit computer running Linux with ≥4 GB RAM

#### Software

The library has been tested with R version 3.5.2 and the R libraries parallel 3.5.2, ggplot2 3.1.0, sets 1.0‐18, arules 1.6‐3, and ggrepel 0.8.0

1 Run R from the command line:
$ R
2 Follow Basic Protocols 1, 2, and 3 to compute beta table bt, ploidy table pl, and DNA admixture table adm, respectively.
3 Given beta table bt, ploidy table pl, and DNA admixture table adm, compute the SCNA clonality table:
> clonality_tb <‐ compute_scna_clonality_table(bt, pl, adm)

## Basic Protocol 6 COMPUTING SINGLE‐NUCLEOTIDE VARIANT CLONALITY

**Figure 7:**
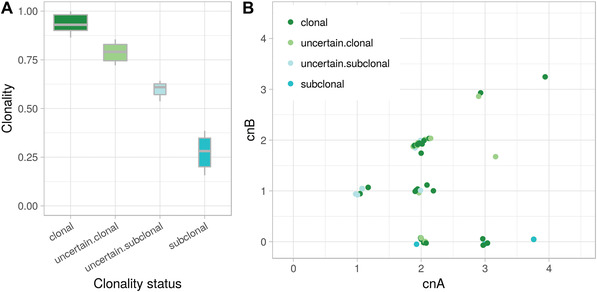
Example of clonality analysis of SNVs. (**A**) Box plot reporting the clonality values of the SNVs of a tumor sample. The clonality value (*y* axis) distributions are shown including all variants of a tumor sample, stratified by the automatically assigned clonality status class (*x* axis). (**B**) For each SNV in panel A, allele‐specific copy‐number data of the genomic segment containing the mutations are reported.

Each SNV is characterized by the variant allele fraction (VAF), i.e., the proportion of reads supporting the alternative allele; intuitively, the VAF is representative of the amount of tumor DNA harboring the mutation (as no alternative read is expected from the admixed normal cells). Therefore, low VAF values correspond to low clonality. In a 100% pure diploid sample, a clonal monoallelic SNV within a wild‐type genomic segment is expected to show a VAF of 0.5 (for simplicity, we here ignore the reference mapping bias; Degner et al., 2009) whereas, in the same setting, an SNV that is present in the 60% of the tumor cells is expected to show a VAF of 0.3. However, several technical and biological factors influence VAF value, including DNA admixture, ploidy, and somatic copy‐number status. In Faltas et al. (2016), we extended the original implementation to deal with SNVs in the context of allele‐specific copy number. SNV VAF ranges over a finite set of values dictated by the DNA copy‐number state: for instance, a clonal SNV in a copy number aberrant segment (CN = 3) in a 100% pure diploid sample may have VAF equal to ⅓, ⅔, or 1, depending on the number of alleles harboring the mutation. By utilizing the sample admixture estimate and the its lower and upper bounds (function compute_dna_admixture), we first estimate the minimum and maximum clonality and next, as for SCNA, assign a discretize clonality value (clonal, uncertain.clonal, uncertain.subclonal, or subclonal). Figure 7A shows an example of SNV clonality (*y* axis) distributions per discretized class (*x* axis) regardless of the copy number of the genomic segments harboring the SNVs (Fig. 7B). Given a tumor sample, the function compute_snv_clonality takes as input the following parameters:

- snv_read_count: a table reporting in each row the genomic coordinates of an SNV together with the numbers of reference and alternative reads covering the mutated position;
- beta_table: a table created using the function described in Basic Protocol 1;
- ploidy_table: a table created using the function described in Basic Protocol 2;
- admixture_table: a table created using the function described in Basic Protocol 3;
- error_tb: the same error_tb used in the function compute_dna_admixture of Basic Protocol 3; error around beta is propagated to assess clonality estimate boundary and in turn is used for its discretization;
- error_rate (default = 0.05): fraction of SNVs to exclude based on adjusted VAF distribution.

The function compute_snv_clonality extends the input table snv_read_count with clonality‐related columns:

- cnA, cnB: allele‐specific copy numbers of the genomic segment containing the SNV;
- t_af_corr: tumor VAF adjusted for ploidy, admixture, and allele‐specific copy number;
- SNV.clonality: percentage of tumor cells harboring the SNV;
- SNV.clonality.status: discretized SNV.clonality.

### Necessary Resources

#### Hardware

64‐bit computer running Linux with ≥4 GB RAM

#### Software

The library has been tested with R version 3.5.2 and the R libraries parallel 3.5.2, ggplot2 3.1.0, sets 1.0‐18, arules 1.6‐3, and ggrepel 0.8.0

1 Run R from the command line:
$ R
2 Follow Basic Protocols 1, 2, and 3 to compute beta table bt, ploidy table pl, and DNA admixture table adm, respectively.
3 Read an SNV table snv_reads with columns rc_ref_tumor and rc_alt_tumor for reference and alternative read counts, respectively:
> read.table(system.file(“sample_snv_read_count.tsv”, package = “CLONETv2”),header = T, as.is=T, comment.char = “", check.names = F, na.strings = ”‐")
4 Given beta table bt, ploidy table pl, and DNA admixture table adm, compute the clonality of SNVs:
> snv_clonality_tb <‐ compute_snv_clonality(“sample1”, snv_reads, bt, pl, adm)

## GUIDELINES FOR UNDERSTANDING RESULTS

**Figure 8:**
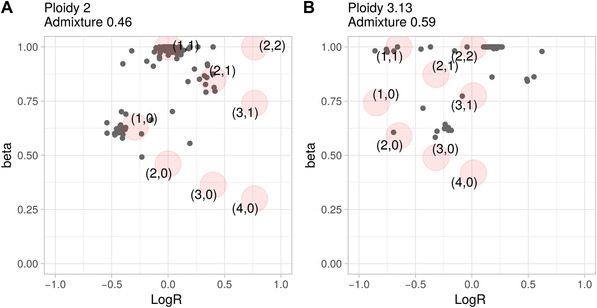
Example of beta vs. logR of segments obtained with different segmentation algorithms. Plots of beta vs. logR for the tumor sample from Figure 2A based on the logR values produced with EXCAVATOR2 (**A**) or FACETs (**B**) are shown. Gray dots represent genomic segments, and light red circles represent expected (beta, logR) values corresponding to the estimated ploidy and DNA admixture (reported above the plots).

We present a complete R package to compute allele‐specific data from next‐generation sequencing experiments with paired tumor and matched normal DNA samples. CLONETv2 works on preprocessed data (not on BAM or fastq files), including segmented genomic profiles and pileups of relevant genomic positions. This makes CLONETv2 more flexible than tools such as ABSOLUTE (Carter et al., 2012), which requires segmented data from HAPSEG (bundled with ABSOLUTE), or FACETS (Shen & Seshan, 2016), which integrates logR segmentation with allele‐specific analysis. The advantage is that CLONETv2 allows the user to choose the segmentation solution that best fits the study needs. As a didactic example, we ran CLONETv2 Basic Protocols 1 to 3 on the sample from Figure 4A (showing segments from CNVkit; Talevich, Shain, Botton, & Bastian, 2016), using segmented data computed with EXCAVATOR2 (D'Aurizio et al., 2016; Fig. 8A) or FACETS (Fig. 8B**)**. EXCAVATOR2 and CNVkit data in this space are similarly distributed, although the former shows noisier signal, and the ploidy and DNA admixture estimates perfectly match. In contrast, with this specific example, the FACETS estimates are different, as expected given, for instance, a set of segments with logR around –0.75 and beta = 1.

**Figure 9:**
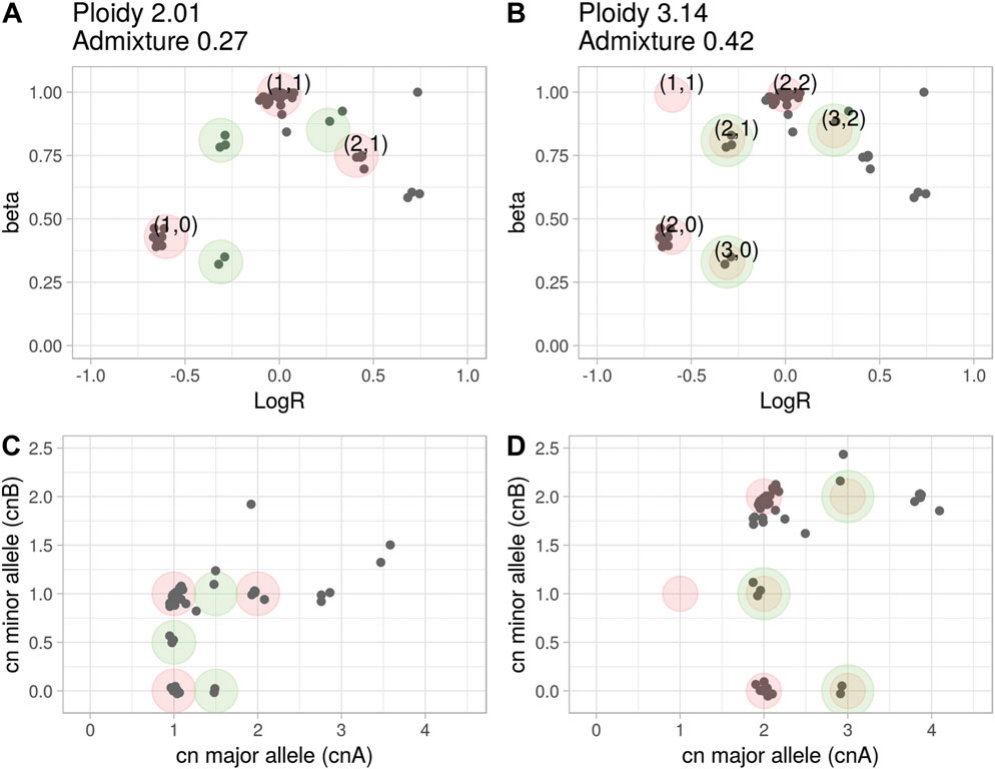
Example of conflicting ploidy estimates. Beta‐vs.‐logR plot of the same tumor sample based on two sets of estimates for ploidy and DNA admixture. Panels **A** and **B** show expected positions for different allele‐specific copy number based on each set of ploidy and DNA admixture estimates. Gray dots represent genomic segments, light red circles represent expected (beta, logR) values corresponding to the estimated ploidy and DNA admixture (reported above the plots), green circles (A) highlight genomic segments for which the estimates do not fit the observed values, and light red circles with green borders in panel B correspond to green circles in panel A. Panels **C** and **D** show allele‐specific copy‐number plots given the estimates in A and B, respectively. Circle color codes are as for panels A and B.

The central notion introduced with CLONETv2 is the proportion of neutral reads beta calculated in Basic Protocol 1. This value expands the one‐dimensional logR space returned by the segmentation algorithms to the two‐dimensional beta‐vs.‐logR space; an example of the utility of this approach is offered in Figure 3B, in which CLONETv2 resolves an ambiguous logR profile from by utilizing beta values (Support Protocol 2). However, as more complex genomic profiles may require inspection of output estimates, we designed a function, check_ploidy_and_admixture, to help the user interpret complex copy‐number data. Figure 9A shows a beta‐vs.‐logR plot of a sample, S, that CLONETv2 defines as having ploidy equals = 2.01 (diploidy) and low DNA admixture. The unique feature of the check_ploidy_and_admixture function is its ability to plot the expected position of a genomic segment in the beta‐vs.‐logR space, given ploidy and DNA admixture (red circles). In Figure 9A, green circles highlight genomic segments that are not explained by estimated ploidy and DNA admixture and are compatible with subclonality, as in Figure 6. However, an alternative interpretation is possible, whereby sample S is aneuploid, and no wild‐type segments are present throughout the tumor genome; the segments in (1, 0) (Fig. 9A) instead represent CN‐LOH (as depicted in Fig. 9B, due to a shift in the logR signal; Basic Protocol 2) and, therefore, wild‐type segments are expected at coordinates (–0.67, 1). Applying the log shift equation (Basic Protocol 2) results in a ploidy of 3.14, and the function compute_dna_admixture, in turn, estimates a DNA admixture value of 0.42. Subclonal copy‐number segments (green circles, Fig. 9A) are then classified as clonal (red circles with green border, Fig. 9B). Given the observed data, both interpretations are plausible. The allele‐specific plots (Figs. 9C and 9D for Fig. 9A and B, respectively), transparent to ploidy and DNA admixture values, may provide additional information to contextualize the two scenarios. The first one (Fig. 9C) represents a tumor in which exactly half of the cells harbor exactly the same set of subclonal hemizygous deletions, subclonal CN‐LOH, and subclonal gain (green circles). The second one (Fig. 9D) suggests genomic events that included whole‐genome duplication (or duplication of several chromosomal arms), exemplified by numerous allele‐specific copy numbers of (2, 2) and CN‐LOH (2,0).

Importantly, CLONETv2 computations are agnostic to gene models so as to avoid cross‐study constraints. To facilitate gene‐level‐focused analysis, the outputs of the functions compute_allele_specific_scna_table and compute_scna_clonality_table can be lifted using any gene model that includes chromosome and start and end position information; tables reporting allele‐specific copy number and clonality values are compatible with BED format (Quinlan & Hall, 2010) and can easily be annotated with common gene models from, e.g., Ensembl (Zerbino et al., 2018).

## COMMENTARY

### Background Information

Tumor ploidy and normal DNA admixture fraction are critical parameters in cancer genomic analysis, as incorrect estimation of either one may compromise any downstream analysis (see example in Fig. 9). CLONETv2 provides a reliable and flexible environment to process matched tumor and normal samples together using the function check_ploidy_and_admixture, which help users evaluate the reliability of estimates. Of note, CLONETv2 is bound neither to a specific copy‐number caller nor to specific gene models. Finally, CLONETv2 is distributed as an R package, and thus downstream processing, including allele‐specific copy‐number and subclonality estimation, can be easily integrated into broader analysis pipelines.

### Critical Parameters

CLONETv2 default parameters have been tested in a variety of studies spanning tissue and plasma samples in different tumor settings. However, data analysis from specific experimental conditions or analysis prerequisites would benefit from tweaking CLONETv2 parameters. The parameter min_coverage is common to many CLONETv2 functions and is used to filter out genomic segments with low mean coverage at informative SNPs; min_required_snps filters out segments with too few informative SNPs. Higher values of min_coverage and min_required_snps correspond to more reliable results but at the same time to fewer segments to be used in computing allele‐specific copy number and clonality. The optimal trade‐off between the reliability and extensiveness of the analysis is study dependent. For instance, an ultra‐deep‐sequencing experiment (e.g., mean coverage > 5000×) would benefit from min_coverage higher than 20 (the default value); in fact, that value corresponds to 0.4% of the expected coverage for a sequencing study of that depth and can hardly be distinguished from the background experimental noise. In contrast, low‐pass whole‐genome sequencing experiments (coverage >4×) require a lower min_coverage by design.

A second critical parameter is error_table, a table reporting the error around beta estimates for different combinations of coverage and number of informative SNPs. CLONETv2 has an error table bundled in, obtained by simulating different inputs to the function compute_beta_table with combinations of values for the coverage and the number of informative SNPs. If, for a given genomic segment, the number of informative SNPs and the mean coverage are not reported in the error_table, CLONETv2 uses the nearest available pair of values, as previously described (Prandi et al., 2014).

### Troubleshooting

CLONETv2 offers a robust framework for the genomic analysis of somatic copy‐number data together with the possibility of manually curating estimates (see Support Protocol 2). However, some specific cases may prevent CLONETv2 from completing the analysis.

**Figure 10:**
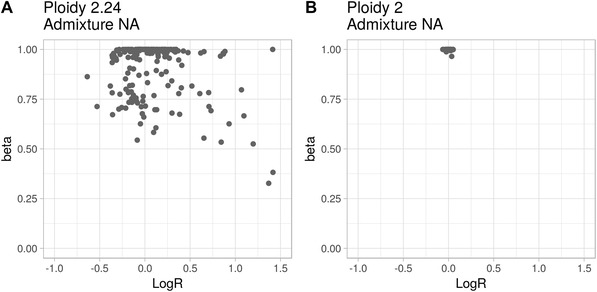
Example of samples with no CLONETv2 DNA admixture estimates. Examples of tumor samples in the beta‐vs.‐logR spaces showing poor segment clusters (**A**) or lack of somatic copy‐number aberrations (**B**).

Figure 10A shows the beta‐vs.‐logR plot of a tumor sample with an uncommon profile. The profile presents genomic segments with all beta values close to 1 (alleles equally represent the parental chromosomes of origin) and logR ranges in the interval (–0.5, 0.5), corresponding to approximately the loss of half a copy and the gain of one copy. Moreover, the cloud of beta values around 0.75 within the same logR range does not fit any CLONETv2 model. These data are either the result of uneven sequence‐read coverage (Wang, Shashikant, Jensen, Altman, & Girirajan, 2017) that affects both the logR signal and the AF of informative SNPs, or the representation of a large number of subclonal populations with diverse ploidy and somatic copy‐number profiles. Altogether, the information from the segmented data and pileup of informative SNPs are not sufficient to disentangle such cases, and such data should not be included in any downstream analysis.

A second problematic case is presented in Figure 10B. All segments show logR around 0 and beta close to 1, i.e., all genomic segments have wild‐type copy number. These beta‐vs.‐logR profile data are compatible with two very different situations: (i) a copy‐number‐quiet tumor sample, i.e., one in which no deletions or amplifications are detected; (ii) a near 100% DNA‐admixed tumor sample, i.e., one in which almost all the cells are non‐tumor cells. The first interpretation points to a potentially interesting case, whereas the second highlights limitations either in the sample of origin or in the preparation. As for the case in Figure 10A, CLONETv2 cannot distinguish between the two interpretations and therefore the sample should not be considered.
